# Artificial intelligence chatbot vs pathology faculty and residents: Real-world clinical questions from a genitourinary treatment planning conference

**DOI:** 10.1093/ajcp/aqae078

**Published:** 2024-06-28

**Authors:** Matthew X Luo, Adam Lyle, Phillip Bennett, Daniel Albertson, Deepika Sirohi, Benjamin L Maughan, Valarie McMurtry, Jonathon Mahlow

**Affiliations:** Department of Pathology, University of Utah, Salt Lake City, UT, US; Department of Pathology, University of Utah, Salt Lake City, UT, US; Department of Pathology, University of Utah, Salt Lake City, UT, US; Department of Pathology, University of Utah, Salt Lake City, UT, US; Departments of Pathology and Laboratory Medicine, University of California, San Francisco, San Francisco, CA, US; Department of Medical Oncology, University of Utah, Salt Lake City, UT, US; Department of Pathology, University of Utah, Salt Lake City, UT, US; Department of Pathology, University of Utah, Salt Lake City, UT, US

**Keywords:** artificial intelligence, natural language processing, genitourinary pathology, treatment planning conference

## Abstract

**Objectives:**

Artificial intelligence (AI)–based chatbots have demonstrated accuracy in a variety of fields, including medicine, but research has yet to substantiate their accuracy and clinical relevance. We evaluated an AI chatbot’s answers to questions posed during a treatment planning conference.

**Methods:**

Pathology residents, pathology faculty, and an AI chatbot (OpenAI ChatGPT [January 30, 2023, release]) answered a questionnaire curated from a genitourinary subspecialty treatment planning conference. Results were evaluated by 2 blinded adjudicators: a clinician expert and a pathology expert. Scores were based on accuracy and clinical relevance.

**Results:**

Overall, faculty scored highest (4.75), followed by the AI chatbot (4.10), research-prepared residents (3.50), and unprepared residents (2.87). The AI chatbot scored statistically significantly better than unprepared residents (*P* = .03) but not statistically significantly different from research-prepared residents (*P* = .33) or faculty (*P* = .30). Residents did not statistically significantly improve after research (*P* = .39), and faculty performed statistically significantly better than both resident categories (unprepared, *P* < .01; research prepared, *P* = .01).

**Conclusions:**

The AI chatbot gave answers to medical questions that were comparable in accuracy and clinical relevance to pathology faculty, suggesting promise for further development. Serious concerns remain, however, that without the ability to provide support with references, AI will face legitimate scrutiny as to how it can be integrated into medical decision-making.

**Key Points**

- Artificial intelligence (AI), a relatively new area of study, has been scrutinized in its ability to provide accurate, clinically relevant answers to clinical questions.
- Although AI chatbots are not as effective as pathology faculty in answering treatment planning conference questions, they still produce fairly accurate and clinically relevant responses.
- AI chatbots have the potential to be useful in answering pathology-related clinical questions.

## Introduction

Artificial intelligence (AI)–based technologies continue to advance across several domains. Natural language processing (NLP) is an area where computational algorithms digest word-based information with a specific output goal in mind using a large language model. Multiple companies have launched chatbots that use NLP approaches to simulate and advance conversational dialogue with humans. These chatbots have variable levels of sophistication, but 1 platform, Chat Generative Pre-Trained Transformer (ChatGPT), created by OpenAI,^1^ has moved to the forefront of international attention. ChatGPT is an NLP application in the form of a chatbot trained using a method called Reinforcement Learning with Human Feedback. Although ChatGPT was not specifically built to assist in diagnosis of disease, it has a range of potential future applications in the practice of medicine and has demonstrated expertise in many of them, such as drawing clinical correlations to pathologic diagnoses, improving patient care, answering “board-style” questions, and providing personalized advice.^2-4^

As with any emerging technology, however, there are challenges to overcome before AI chatbots can be widely adopted in health care settings. Current research has yet to substantiate their accuracy and clinical relevance. This study sought to evaluate AI’s ability to answer real pathology-related clinical questions asked during a treatment planning conference.

## Methods

Questions asked at a genitourinary (GU) subspecialty treatment planning conference were collected and synthesized into a 5-item questionnaire. This questionnaire was answered by 5 participants, including 3 pathology residents and 2 pathology faculty, as well as an AI chatbot (ChatGPT [January 30, 2023, edition]). The following questions of varying complexity were asked of the participants:

- “Is the loss of PAX8 expression unusual in metastatic [renal cell carcinoma] with a biopsy-confirmed kidney primary?”
- “Is it unusual for tubulocystic [renal cell carcinoma] to metastasize?”
- “In the oligometastatic setting with an inconclusive bone biopsy, would biopsy of the presumed primary tumor yield more diagnostic results?”
- “What is the clinical significance of cystic trophoblastic tumor in patients with testicular cancer?”
- “Are two atypical glands sufficient for the diagnosis of prostatic adenocarcinoma and pelvic radiation?”

The residents first answered the questionnaire without preparation, and then repeated it after a brief period of research. The results were evaluated by 2 adjudicators blinded to answer sources: a clinician expert (academic medical oncologist with >5 years of GU-specific experience) and a pathology expert (academic pathologist with >10 years of GU-specific experience). Scores were evaluated on a scale of 1 to 5 (1 = inaccurate/not clinically relevant, 5 = accurate/clinically relevant) and analyzed using the Kruskal-Wallis statistical method. Standardized answers—those that received perfect scores of 5/5 by both adjudicators—are included in Supplemental Materials 1 (all supplementary material is available at *American Journal of Clinical Pathology* online).

## Results

Average scores varied among participants Table 1. Residents without preparation scored an average of 2.87, a score that improved after a brief period of research to 3.50. Faculty scored an average of 4.75, and the AI chatbot scored an average of 4.10.

**TABLE 1:** Questionnaire Response Scores

Adjudicator	Question	Artificial intelligence	Faculty 1	Faculty 2	Research-prepared resident 1	Research-prepared resident 2	Unprepared resident 1	Unprepared resident 2	Unprepared resident 3
Clinician	1	5	5	5	3	1	2	3	3
Clinician	2	5	5	5	4	2	1	3	3
Clinician	3	3	5	5	4	1	3	3	3
Clinician	4	4	5	3	4	2	2	1	2
Clinician	5	4	5	3	5	2	3	2	3
Pathologist	1	3	5	5	4	4	4	4	4
Pathologist	2	4	5	5	4	4	2	4	4
Pathologist	3	5	5	5	4	3	4	4	4
Pathologist	4	4	5	5	5	5	1	1	3
Pathologist	5	4	5	4	4	5	4	3	3
Average		4.10	5.00	4.50	4.10	2.90	2.60	2.80	3.20
Combined average		4.10	4.75		3.50		2.87		

**FIGURE 1:**
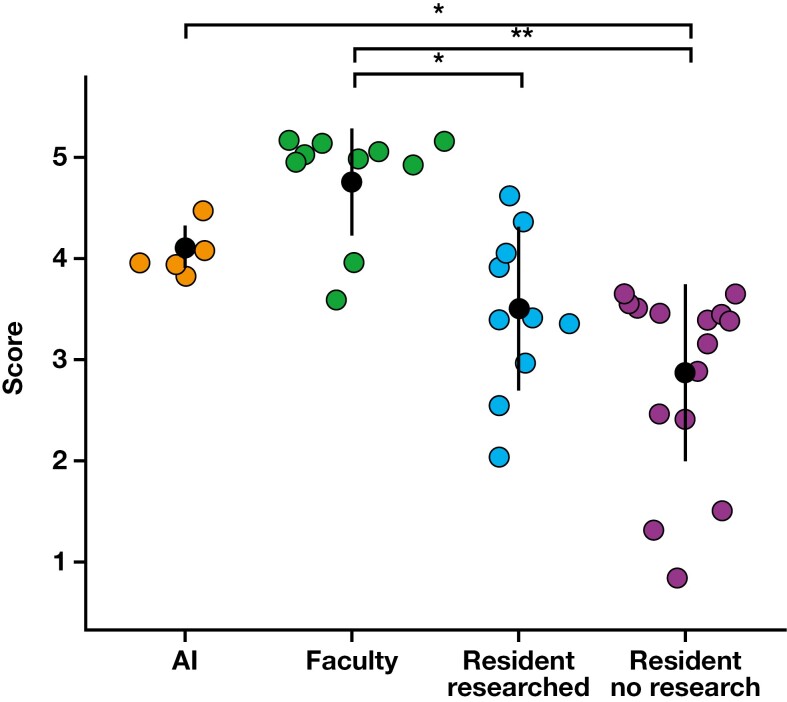
Questionnaire results. AI, artificial intelligence. *P < .05; **P < .01.

Average scores were compared between groups Figure 1. Although residents’ scores improved after the brief research period, this change was not statistically significant (*P* = .39). Although residents without preparation scored statistically significantly worse than both the AI chatbot and faculty (*P* = .03 and *P* < .01, respectively), residents who did research did not perform statistically significantly worse than the AI chatbot (*P* = .33). Residents who did research still performed statistically significantly worse than faculty (*P* = .01), however. Average faculty scores were higher than those of the AI chatbot, but this difference was not statistically significant (*P* = .30).

## Discussion

In this study, AI’s ability to answer treatment planning conference questions both accurately and with clinical relevance was evaluated. Compared with research-prepared pathology residents and pathology faculty, the AI chatbot was able to answer medical questions with comparable accuracy and clinical relevance. The questions with differing levels of complexity served to test the ability of ChatGPT to answer nuanced clinical questions, most of which do not have a simple yes or no answer. Although AI NLP is still a relatively new field of study, these results show that there is potential for further developments as the technology continues to improve.

Although AI is still a relatively new technology, its credibility in making important medical decisions remains a serious and legitimate concern. The field of medicine is empirically evidence based, using the best available evidence from scientific research to make decisions for patient care. Machine learning can improve accuracy over time, but expert supervision and validation of sources are necessary to ensure that the information chatbots provide is correct. Given the lack of transparency in terms of which data sets the AI system is analyzing, NPL chatbots such as ChatGPT currently do not fulfill the requirements necessary for integration into medical decision-making. Whether the answers provided are correct is inconsequential: the risk of taking an answer at face value that determines patient care without reliable sources is too great.

Moreover, the body of research on the efficacy of NLP chatbots such as ChatGPT is still limited, and further independent, large-scale studies and consensus among the medical community are required to assess their validity. Currently, chatbots cannot take the place of expert physicians; the complexity and heterogeneity of disease requires individualized management using the most up-to-date data to provide sophisticated and rationalized care. Chatbots may develop the ability to analyze and synthesize the available literature, but the interpretation of that literature must be left to human physicians, who can validate the accuracy of the information provided using trusted resources. More recent literature may debunk “proven facts” stated by previous research papers, but it is uncertain which of these answers the chatbot will provide or how AI-based systems resolves discrepancies.

The limitations of this study are acknowledged, such as a small sample size and subjective scoring by adjudicators. That said, it still reflects real-life decision-making processes in medicine and at treatment planning conferences.

## Supplementary Material

aqae078_suppl_Supplementary_Material
