# Supplementary material for: Artificial intelligence chatbot vs pathology faculty and residents: Real-world clinical questions from a genitourinary treatment planning conference
Source: Am J Clin Pathol. 2024 Jun 28;162(6):541–3. doi: 10.1093/ajcp/aqae078 (PMC11637512; doi:10.1093/ajcp/aqae078)
Supplement: aqae078_suppl_Supplementary_Material [file aqae078_suppl_supplementary_material.docx]

Supplemental Materials:

Standardized answers to GU TPC Questions. These answers were deemed accurate and highly clinically relevant/helpful with scores of 5/5 in the opinion of both adjudicators.

**1. Is the loss of PAX8 expression unusual in metastatic RCC with a biopsy confirmed kidney primary?**

PAX8 is an excellent although not entirely specific IHC stain for kidney lineage, its loss would be very unusual, unless the metastatic tumor had dedifferentiated, and may suggest the need for additional imaging to exclude a second primary. It would be helpful to know if the underlying tumor had high grade features or sarcomatoid differentiation as PAX8 loss may be more likely in those situations.

**2. Is it unusual for tubulocystic RCC to metastasize?**

Tubulocystic RCC generally behaves in an indolent fashion and a metastasis is quite unusual, although admittedly well described in literature. That said, tubulocystic RCC is also an uncommon diagnosis on its own and review of the underlying pathologic diagnosis for accuracy/agreement should be considered given this unexpected clinical behavior.

**3. In the oligometastatic setting with an inconclusive bone biopsy would biopsy of the presumed primary tumor yield more diagnostic results?**

From the pathologist’s perspective, the primary lesion will likely yield more representative and helpful information. Biopsy of the primary organ allows comparison to background tissue, inspection for a co-existing in-situ component, and may also show tumor heterogeneity that may explain unusual metastatic presentations or high grade / dedifferentiated components.

**4. What is the clinical significance of cystic trophoblastic tumor in patients with testicular cancer?**

Cystic trophoblastic tumor is a rare although increasingly recognized diagnosis in the retroperitoneal lymph nodes of patients with treated metastatic choriocarcinoma. From a clinical perspective cystic trophoblastic tumor has the same significance as metastatic teratoma, it is unlikely to exhibit the same aggressive clinical features as choriocarcinoma. Management should be surgical (if not completely resected) or surveillance. Continued monitoring of serum HCG is not unreasonable given the morphologic overlap with choriocarcinoma.

**5. Are two atypical glands sufficient for the diagnosis of prostatic adenocarcinoma and pelvic radiation?**

No. Most GU pathologists use Epstein criteria for the diagnosis of prostatic carcinoma, which suggests 3 or more atypical glands and only then with absolutely all and perfect histologic criteria met. In this scenario, most pathologists would diagnose atypical small acinar proliferation even with immunohistochemical support. This is simply too little tumor for a definitive diagnosis of carcinoma, particularly in the context that this patient would not be managed with active surveillance since radiation is being considered.
